# Testing Front-of-Package Labels on Packaged Food for Adult and Adolescent Population in Bangladesh

**DOI:** 10.1016/j.cdnut.2026.109464

**Published:** 2026-07-29

**Authors:** Abu Ahmed Shamim, Lindsey Smith Taillie, Oumma Halima, Nisarga Bahar, Md Hafizul Islam, Md Mokbul Hossain, Sneha Sarwar, Ahmed K Abrar, Ummay Afroza, Sohel Reza Choudhury, Lindsay Steele, Nazma Shaheen

**Affiliations:** 1 Centre for Non-communicable Diseases and Nutrition, BRAC James P Grant School of Public Health, BRAC University, Dhaka, Bangladesh; 2 Department of Nutrition, University of North Carolina at Chapel Hill, Chapel Hill, NC, United States; 3 Institute of Nutrition and Food Science, University of Dhaka, Dhaka, Bangladesh; 4 National Heart Foundation of Bangladesh, Dhaka, Bangladesh; 5 Resolve to Save Lives, Alexandria, VA, United States

**Keywords:** front-of-pack labeling, packaged foods, warning labels, nutrition labeling, nutrients of concern, adolescents and adults, randomized controlled trial, Bangladesh

## Abstract

**Background:**

Front-of-pack labeling (FOPL) is a promising tool to improve consumer understanding of unhealthy foods and reduce diet-related non-communicable diseases (NCDs). However, evidence on the effectiveness of different formats in Bangladesh is limited.

**Objectives:**

This study evaluated the impact of 4 FOPL formats—warning label (WL), health star rating (HSR), multiple traffic light (MTL), and guideline daily allowance (GDA)—on consumers’ ability to identify products high in nutrients of concern and their intention to purchase these products.

**Methods:**

A nationally representative randomized controlled trial was conducted across all 8 divisions of Bangladesh among adolescents (10–18 y) and adults (19–60 y). Participants were randomly assigned to 5 arms (control, WL, HSR, MTL, or GDA) and evaluated using mock packaged foods. Primary outcomes included correct identification of foods high in sugar, sodium, saturated fat, or calories and purchase intention. Secondary outcomes included perceived unhealthiness, perceived message effectiveness (PME), and cognitive elaboration. Tertiary outcomes assessed reactions to FOPL formats.

**Results:**

WLs showed the greatest impact on correct product identification among adolescents and adults. Compared with the control (14%–15%), correct identification was significantly higher with WLs (68%–73%) and MTL (62%–65%) (*P* < 0.001). Although MTL outperformed GDA, it was generally less effective than WLs. WLs were most effective at reducing adults’ intention to purchase harmful products. All FOPL formats improved psychological responses compared with control. WLs, followed by MTL, had the strongest effects on perceived unhealthiness, health concern, PME, cognitive elaboration, credibility, and support for labeling, with WLs most strongly discouraging consumption and increasing health concern.

**Conclusions:**

Interpretive FOPL systems outperform noninterpretive labels in improving consumer understanding, with WLs showing the strongest effects. These findings support the use of mandatory WLs as an effective policy to promote healthier food choices and address diet-related NCDs in Bangladesh.

This study was preregistered at the Open Science Framework on February 5, 2025. Registration DOI: https://doi.org/10.17605/OSF.IO/8ZDK6

## Introduction

The rising burden of non-communicable diseases (NCDs) has emerged as an invisible epidemic in low- and middle-income countries (LMICs). Of all of the deaths from NCDs worldwide, nearly three-quarters occur in LMICs, driven by aging populations, unhealthy lifestyles, and increasing consumption of processed and ultraprocessed food products [1]. In Bangladesh, changes in lifestyle, increased purchasing power, and rapid urbanization contributed to a notable shift in dietary preferences toward the consumption of commercially produced packaged foods (hereafter, termed as packaged foods) [2,3].

Recent evidence shows widespread consumption of packaged foods across both rural and urban areas in Bangladesh, with significant variation by age group [4]. The frequency of unhealthy food and beverage consumption among adolescents in Bangladesh is high in both urban and rural areas [5]. The same national survey also reported higher consumption of unhealthy foods and beverages among adults (20–59 y) [6] and elderly people (≥60 y) [7].

Packaged foods are typically high in sugar, sodium, SFAs, and trans fatty acids and are high in energy. Excessive intake of these foods leads to an enhanced risk of overweight and obesity, NCDs, and related mortality [[8], [9], [10]]. In current practices, to protect consumers’ health and safety, food labeling is regulated by the Bangladesh Food Safety Authority, and regulations include nutrition information (total calories, protein, fat, carbohydrates, total sugar, sodium, and salt) per 100 mL or 100 g pack [11]. However, recent evidence indicates that consumers predominantly check the manufacturing or expiry date on packaged foods, while only a small proportion consult the nutrition facts panel, and those who do often face difficulties interpreting the information provided [12]. To help consumers identify unhealthy packaged foods and beverages, front-of-package labels (FOPLs) have been recommended by the WHO [13]. Therefore, introducing FOPLs in Bangladesh is now essential to control the consumption of ultraprocessed foods, overcoming barriers such as illiteracy, low awareness, and distrust.

Existing literature suggests that using a warning label (WL) to inform consumers about unhealthy foods and discourage their consumption is likely to produce the most considerable health benefits. In contrast, there is limited country-specific evidence on the effectiveness of other interpretive FOPLs, such as multiple traffic lights (MTLs), health star ratings (HSRs), and the guideline daily allowance (GDA) [14]. A qualitative study on FOPL was conducted in Bangladesh [15] to assess potential consumers’ reactions to various FOPL formats especially designed for Bangladesh and found that interpretative FOPLs were well understood by consumers, particularly WLs. However, no previous study in Bangladesh has systematically assessed the effect of exposure to various FOPL formats on the ability of consumers to identify foods high in fat, sugar, and sodium (HFSS), a key step on the pathway to reducing consumption. Moreover, experimental studies conducted in other countries such as India [16], South Africa [17], and Kenya [18] did not include adolescents, a population that frequently consumes packaged foods.

The objective of this study was to experimentally test the impact of 4 FOPL formats (WL, HSR, MTL, and GDA) compared with a control label on Bangladeshi adults’ and adolescents’ ability to identify HFSS foods and their intention to purchase these products. In addition, the secondary objective of the study was to study the perceptions of unhealthiness, perceived message effectiveness (PME), and cognitive elaboration. Additional outcomes assessed included participants’ reactions to different FOPL formats.

## Methods

### Institutional review board approvals

This study was reviewed and approved by the Ethics Review Committee of the National Heart Foundation Hospital and Research Institute in Dhaka, Bangladesh (Ref: N.H.F.H. & amp; R.I/4/14/7/Ad-2783) on 29 December 2024. Before starting the interview, the participants were given a short brief of the study and its objectives. Written informed consent was obtained from all adult participants. For adolescents (aged 10–18 y), written assent was obtained from the adolescents themselves, and consent was simultaneously obtained from their legal guardians.

This study was preregistered at the Open Science Framework on February 5, 2025. Registration DOI: https://doi.org/10.17605/OSF.IO/8ZDK6

### Study design

The present study employed a multiarm, individually randomized experimental design to evaluate the impact of 4 different FOPL formats on consumer understanding, perceptions, and purchase intentions of packaged foods. Participants were individually randomly assigned to one of 5 study arms: barcode (control), HSR, WL, GDA, or MTL.

### Study population

The study population comprised adults and adolescents residing in rural and urban Bangladesh. Adults were eligible if they were aged 19 to 60 y and had purchased packaged foods at least once in the past month and were involved in ≥50% of the household grocery purchasing decisions. Eligible adolescents aged 10 to 18 y had also purchased packaged foods at least once in the past month.

Exclusion criteria for both groups included vision impairment. To ensure representativeness, the proportion of primary incomplete participants (0–4 y of education) was capped at 25% of the sample; the final sample included ∼17% such participants; of them, <1% adolescents and <8% adults did not complete any education year. We included them because they are buyers and consumers of processed foods and because it is important for any labeling policy that is developed to work for illiterate populations as well as literate populations. We hypothesized that interpretive FOPLs (WL and MTL) would be appropriate for such participants. This hypothesis was based on the pictorial and color-coded nature of these formats, which are easier to interpret without requiring advanced literacy skills, thereby making them more appropriate for individuals with limited formal education.

### Sample size and sampling strategy

Sample size estimation drew on evidence from a similar Indian trial [16]. Using a 5% type I error rate, 20% type II error rate (80% power), and a design effect of 1.61 [5,19], the required sample size was 600 participants per arm, totaling 3000 per age group.

A 4-stage cluster sampling design was used to obtain a sample across all 8 administrative divisions of Bangladesh (Rajshahi, Rangpur, Barishal, Khulna, Chattogram, Sylhet, Dhaka, and Mymensingh) (Supplemental Figure 1). From each division, one district was randomly selected (Naogaon, Gaibandha, Barishal, Kushtia, Lakshmipur, Moulvibazar, Narayanganj, and Mymensingh). Study locations were selected based on the primary sampling units of the Bangladesh Household Income and Expenditure Survey, ensuring consistency with the national census framework and preserving representativeness across rural and urban settings, ultimately resulting in 45 study site locations (27 rural and 18 urban). Data collection took place between February 28, 2025 and June 2, 2025.

In each study cluster, 250 to 280 eligible individuals (50% adults and 50% adolescents) were listed through household visits. From this list, 68 adults and an equal number of adolescents were randomly selected per cluster, with equal representation of males and females in both age groups. All randomly assigned participants were included in the interview process. In case of nonresponses mainly due to unavailability of the respondent, the study team attempted at least 3 times to meet the participant. The nonresponse or refusal rate was nearly 5% (4–6 nonresponses per cluster). Whenever there was a refusal or nonresponse, the enumerator informed the statistician, and the statistician replaced this from the list of additional potential participants. To minimize allocation bias, participants were randomly assigned to one of 4 FOPL formats or a control condition, ensuring equal distribution across study arms.

### Stimuli

**FIGURE 1:**
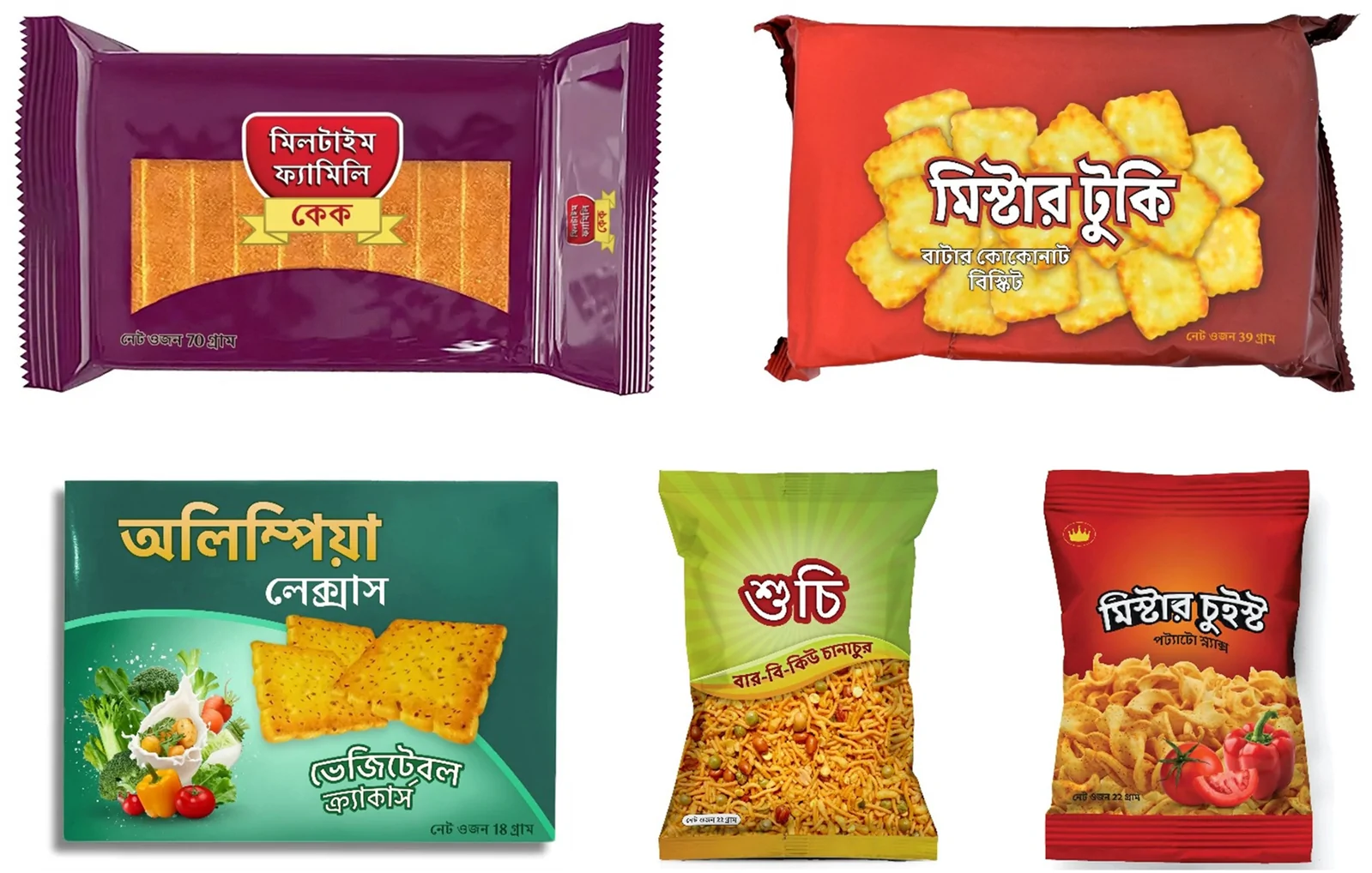
Mock products of 5 commonly consumed packaged foods selected based on prior surveys.

The FOPL formats were displayed on 5 commonly consumed products in Bangladesh [4]: cake, salty biscuit, chips, chanachur (salty snack), and sweet biscuit. A graphic designer developed mock products, and a team of nutritionists created mock nutrient profiles (i.e., calories, sugar, saturated fat, and sodium) for each of the 5 products based on a top-selling commercial brand for each category (Figure 1) [16].

### Labels

Supplemental Table 1 compares 4 FOPL systems (MTL, Summary Indicators, Reference Intake/GDA, and WL) in terms of their design and interpretability. It shows that WL provide the clearest, nutrient-specific information, whereas 3 other formats vary in clarity and may present mixed messages, rely on numerical data, or be more susceptible to industry manipulation.

**FIGURE 2:**
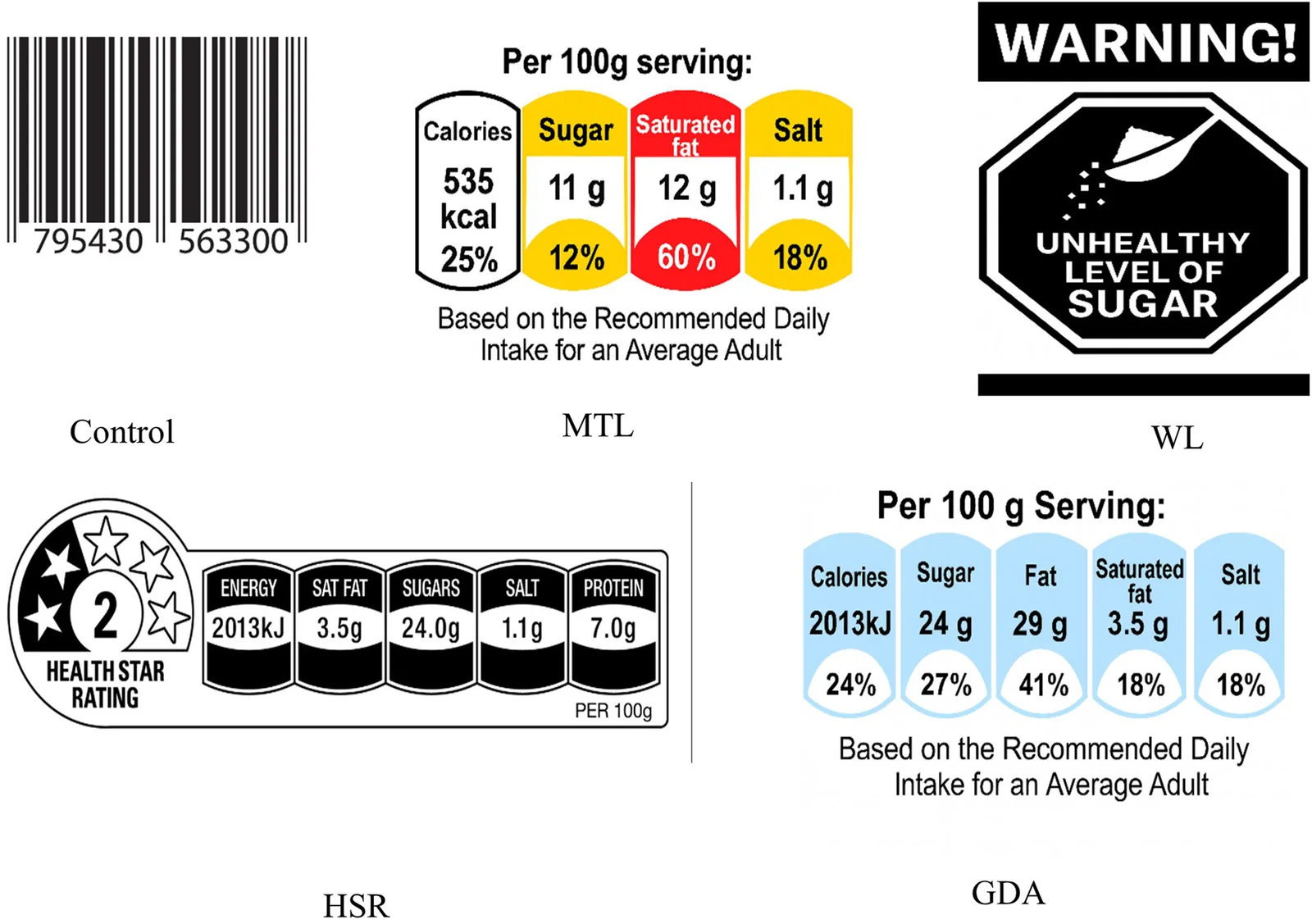
Five types of front-of-package labels used in the study (Control, MTL, WL, HSR, GDA) (English translations of the Bangla versions used in the study). GDA, guideline daily allowance; HSR, health star rating; MTL, multiple traffic light; WL, warning label.

The FOPL formats tested in this study were selected based on qualitative research conducted preceding this randomized controlled trial [15] and were finalized by the Technical Working Group (Figure 2). The study used Bangla language versions of the FOPLs, which are presented in Supplemental Figure 2. Four FOPLs—the WL, MTL, HSR, and the GDA [16]—were selected in addition to the barcode control. A barcode label was used as a control label because it serves as a visual information on the front of the food package while conveying neutral information about the product’s nutritional content. A professional designer developed 5 sets of each mock product containing WL, MTL, HSR, GDA, and a barcode.

### Pilot testing and modification of questionnaire

A pilot test, conducted with 964 adult and adolescent participants across rural and urban sites, assessed the clarity of mock food products, FOPLs, study procedures, and questionnaire items. Insights from this phase led to refinements in the questionnaire, including adding an “I do not know” option for nutrient-identification questions to avoid forcing uninformed responses. Additionally, assets associated with financial status were revised after participants showed confusion with the term “bills,” prompting a shift to more contextually relevant descriptions of household economic conditions.

### Procedure

Participants were randomly assigned to 1 of 5 arms: control label, HSR, WL, GDA, or MTL label using an allocation ratio of 1:1:1:1:1. Participants then viewed images of products in random order with an FOPL on the product according to the assigned arm (as described above). In the control condition, all products had barcode labels. In the HSR condition, all products displayed stars. In the WL condition, products are shown with the relevant warning(s) for sugar, sodium, and/or saturated fat. In the GDA condition, all products had a GDA with the appropriate nutritional information. In the MTL condition, products were shown with MTLs with the applicable color code (green, yellow, or red) for each sugar, saturated fat, and salt.

Interviewers showed participants images of products using an A5-size booklet in random order. They asked them to assess the product based on the nutrients of concern and their reactions to the label. The participants viewed pictures of FOPL formats and answered questions about which label they preferred. All data were entered into a smartphone app (KoboCollect Software) during the interview.

### Outcome measures

#### Primary outcomes

The primary outcomes of the study were as follows: *1*) participants’ ability to correctly identify HFSS products and *2*) intentions to purchase HFSS products. Ability to identify HFSS products was assessed using product-specific questions asking, “Do you think this product has high (nutrient of concern)?” Response options included yes, no, and I do not know. A response was considered correct if it accurately reflected the predefined nutrient profile of the product based on the study’s nutrient thresholds. Responses of “no” or “I do not know” for products that were high in a given nutrient were classified as incorrect.

Intentions to purchase HFSS products were assessed using product-specific questions asking participants whether they would purchase the product in the next week if it were available. Those responding “yes” were further asked to indicate the likelihood of purchase, allowing assessment of both purchase intention and its strength.

#### Secondary outcomes

Secondary outcomes included perceptions of unhealthiness, PME, and cognitive elaboration. Participants were asked, “Is this product unhealthy?” (yes/no). If they answered yes, they were asked, “How unhealthy is it?,” with response options ranging from 1 to 3 (very much, somewhat, very little). For PME, participants were asked whether the label made them feel concerned about the health consequences of consuming the product, made the product seem unpleasant, and made them feel discouraged from wanting to consume the product. For all label assessment items, response options were yes/no, and respondents who answered yes were subsequently asked, “How much?,” with responses ranging from 1 (very much) to 3 (very little). The mean was calculated from responses to the 3 PME items to create a composite PME score when internal consistency was acceptable (Cronbach’s α > 0.70). Cognitive elaboration was assessed by asking whether the label prompted participants to think about health problems associated with consuming the product (yes/no), followed by the extent of that thinking.

#### Tertiary outcomes

Tertiary outcomes assessed participants’ reactions to different FOPL formats. These included perceived label understanding, perceived learning of new information, perceived truthfulness of the label, desire to have the label displayed on products, attention capture, perceived unpleasantness, and concern about health consequences. Participants were also asked to indicate which FOPL format most discouraged product consumption. These outcomes were measured using structured response options and reported as the proportion of participants endorsing each response. These measures were assessed separately among adolescents and adults following exposure to the assigned labeling condition.

### Statistical analysis

In our study, there were no missing values because an enumerator asked participants each question, which prevents participants from skipping items. Descriptive statistics were used to summarize participant characteristics. For all items measured on a Likert scale, responses from the agreement item (yes/no) and the corresponding strength-of-agreement item were combined for each participant to construct a 4-point Likert scale. The resulting scale was subsequently recoded from 1 (“not at all”) to 4 (“very much”) to facilitate more intuitive interpretation. In the main analyses, each FOP label condition was compared with the control arm. Pair-wise comparisons between label conditions were also conducted. Poisson regression was used for comparing proportion (percentage) of responses by study arm to the control condition for binary outcomes (correct identification compared with incorrect identification of nutrients of concern by study arms). This approach was selected because the prevalence of several study outcomes was relatively high (e.g., 73% correct identification among adolescents with WL labels) and prevalence ratios are more interpretable than odds ratios in cross-sectional analyses in such conditions [20], where logistic regression can substantially overestimate the effect size compared with risk ratios. Poisson regression models were also applied in a similar study conducted in South Africa [17] to compare the effects of different FOPL conditions.

For main analysis, results are presented as a percentage of participants with a positive outcome response. For pairwise comparisons, results are presented as relative risk (RR) estimates comparing pairs of label conditions, where an RR > 1 indicates a higher proportion of participants exposed to label X correctly identified products high in nutrients of concern or unhealthy products compared with those exposed to label Y. Moreover, pairwise comparisons of the outcome variable by the sociodemographic characteristics (gender, age, education, area of residence, and financial situation) are presented as percentages.

Although individual randomization was employed, some characteristics were not perfectly balanced, as indicated in Table 1. Therefore, the models were adjusted for potential confounders, including age, sex, level of education, and area of residence. However, the adjusted models yielded almost similar estimates in all cases. The covariate adjustment was not prespecified in the trial registration but was introduced during the analysis phase. Finally, the unadjusted models were used and described in the manuscript (Supplemental Figures 3 and 4). However, in line with CONSORT principles of transparency, the findings of the adjusted models were also presented.

**TABLE 1:** Sociodemographic characteristics of the study participants, *n* (%)

Characteristics	Control *N* = 1230	HSR *N* = 1230	WL *N* = 1244	GDA *N* = 1233	MTL *N* = 1237
**Age (adolescents)**					
10–14 y	310 (50.7)	306 (50.4)	316 (50.6)	321 (51.3)	323 (52.3)
15–18 y	301 (49.3)	301 (49.6)	309 (49.4)	305 (48.7)	295 (47.7)
**Sex (adolescents)**					
Girl	294 (48.1)	302 (49.8)	323 (51.7)	337 (53.8)	285 (46.1)
Boy	317 (51.9)	305 (50.2)	302 (48.3)	289 (46.2)	333 (53.9)
**Education (adolescents)**					
Grade 0–4	174 (28.5)	159 (26.2)	160 (25.6)	168 (26.8)	153 (24.8)
Grade 5	101 (16.5)	81 (13.3)	105 (16.8)	104 (16.6)	102 (16.5)
Grade 6–9	250 (40.9)	293 (48.3)	283 (45.3)	270 (43.1)	289 (46.8)
Grade ≥10	86 (14.1)	74 (12.2)	77 (12.3)	84 (13.4)	74 (12.0)
**Age (adults)**					
19–30	234 (37.8)	230 (36.9)	218 (35.2)	194 (32.0)	244 (39.4)
31–40	210 (33.9)	202 (32.4)	195 (31.5)	234 (38.6)	195 (31.5)
41–50	123 (19.9)	133 (21.3)	137 (22.1)	124 (20.4)	114 (18.4)
51–60	52 (8.4)	58 (9.3)	69 (11.1)	55 (9.1)	66 (10.7)
**Sex (adults)**					
Male	311 (50.2)	286 (45.9)	335 (54.1)	297 (48.9)	312 (50.4)
Female	308 (49.8)	337 (54.1)	284 (45.9)	310 (51.1)	307 (49.6)
**Education (adults)**					
Grade 0–4	88 (14.2)	104 (16.7)	128 (20.7)	103 (17.0)	109 (17.6)
Grade 5	176 (28.4)	181 (29.1)	161 (26.0)	200 (32.9)	154 (24.9)
Grade 6–9	172 (27.8)	175 (28.1)	161 (26.0)	164 (27.0)	194 (31.3)
Grade ≥10	183 (29.6)	163 (26.2)	169 (27.3)	140 (23.1)	162 (26.2)
**Area of residence**					
Rural	738 (60.0)	740 (60.2)	744 (59.8)	740 (60.0)	740 (59.8)
Urban	492 (40.0)	490 (39.8)	500 (40.2)	493 (40.0)	497 (40.2)
**Household financial situation**					
I can meet necessities and buy some luxury items with my income	287 (23.3)	305 (24.8)	312 (25.1)	283 (23.0)	320 (25.9)
I can only meet my necessities with my income	728 (59.2)	717 (58.3)	732 (58.8)	724 (58.7)	706 (57.1)
To meet my necessities, I have to take loans, I cannot buy the necessary stuff	215 (17.5)	208 (16.9)	200 (16.1)	226 (18.3)	211 (17.1)

Abbreviations: GDA, guideline daily allowance; HSR, health star rating; MTL, multiple traffic light; WL, warning label.

Consistent with prior studies, design weights were not required to be incorporated into the analysis, as the primary objective was to estimate the effect of FOPLs compared with a control label, rather than to produce nationally representative estimates of population-level indicators [21,22]. In addition, clusters of approximately equal size were employed, and an equal number of participants was randomly selected for each arm from the designated clusters. However, we replicated the analyses both with and without adjustment for clustering effects. The variation in risk ratio estimates was <5% in most cases, and the direction of association as well as the statistical significance levels remained unchanged. This approach enhanced the likelihood of obtaining unbiased estimates.

Although stratified analyses of the outcomes by sociodemographic characteristics were not planned during the preregistration process of the trial, we conducted exploratory stratified analyses and presented them as supplementary materials to understand whether the impact of the labels differed according to key sociodemographic variables, including gender, age, urban compared with rural, and household financial situation. Education was originally specified as a 4-level variable (with stratified analyses for each level): grade 0 to 4, grade 5, grade 6 to 9, and grade ≥10. We were also interested in assessing whether effects were moderated by literacy; however, we did not directly measure literacy. Thus, we used an education level of grade 0 to 4 as a proxy for illiteracy and conducted an additional stratified analyses for grade 0 to 4 compared with all higher grades.

All the statistical analysis was conducted using the STATA software version 18.

## Results

### Sociodemographic characteristics of the study participants

Table 1 presents the sociodemographic characteristics of participants across the 5 experimental arms (control, HSR, WL, GDA, and MTL), each comprising ∼1230 to 1244 respondents. The study arms were largely comparable across key characteristics (age, sex, and area of residence). Adult educational attainment differed modestly across arms, whereas adolescent education levels were comparable. Baseline purchase behavior was comparable across arms for all products except chips among adults (Supplemental Table 2).

### Correct identification of the nutrient of concern

**FIGURE 3:**
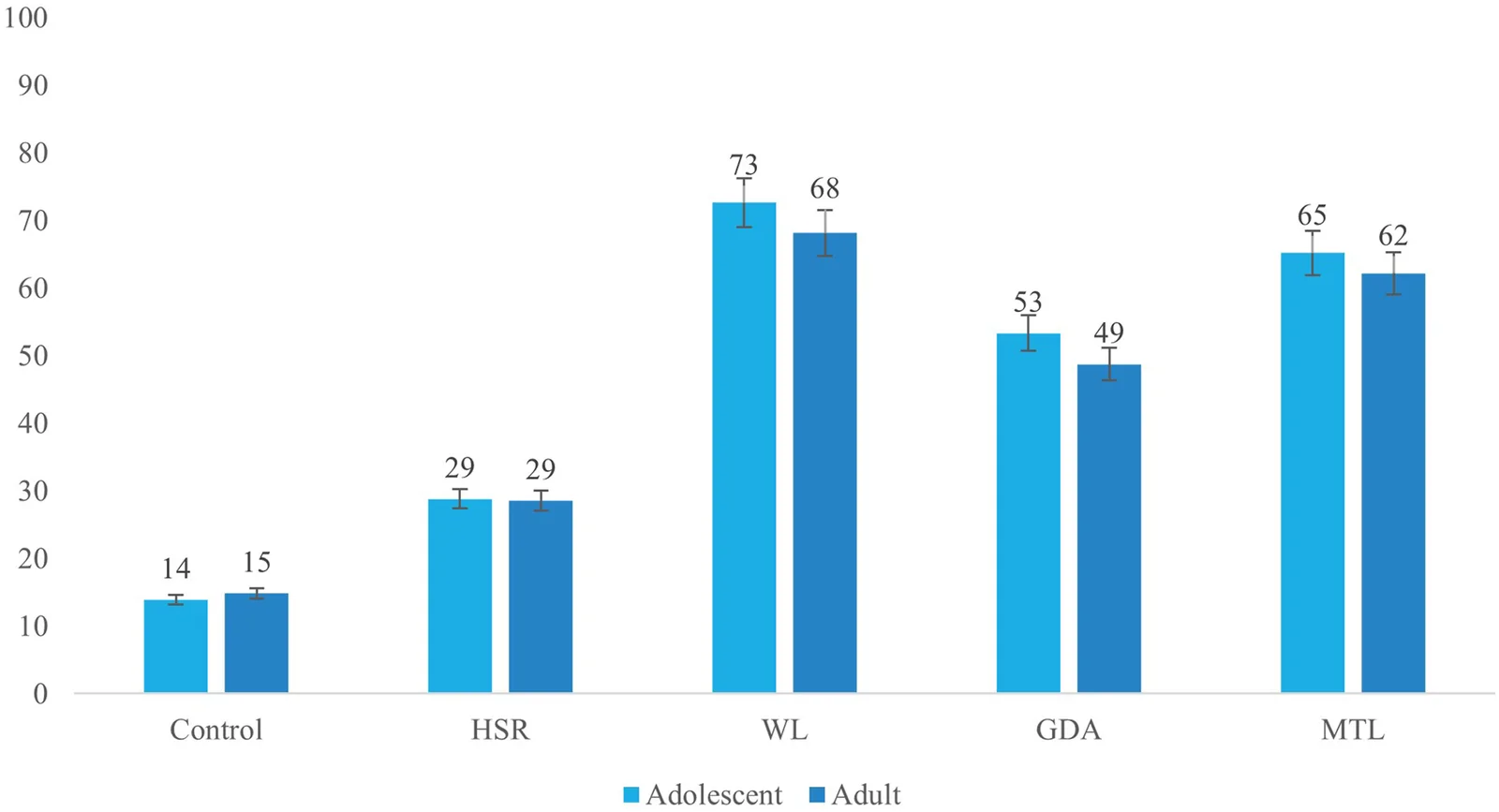
Percent of participants who correctly identified that products were high in nutrient(s) of concern by study arm. GDA, guideline daily allowance; HSR, health star rating; MTL, multiple traffic light; WL, warning label.

In the control (barcode) arm, correct identification of nutrients of concern (HFSS) was low among both adolescents (14%; 95% CI: 11.4, 16.9) and adults (15%; 95% CI: 12.3, 17.9) (Figure 3). All FOPL formats significantly improved the accuracy of correct identification compared with the control (*P* < 0.001 for all comparisons). WL demonstrated the highest effectiveness, with 73% (95% CI: 69.0, 76.0) of adolescents and 68% (95% CI: 64.4, 71.7) of adults correctly identifying unhealthy products. MTL labels ranked second, achieving correct identification rates of 65% (95% CI: 61.4, 68.9) among adolescents and 62% (95% CI: 58.3, 65.9) among adults. GDA labels resulted in correct identification among 53% (95% CI: 49.4, 57.2) of adolescents and 49% (95% CI: 44.8, 52.7) of adults, whereas HSR labels showed more modest improvements at 29% (95% CI: 25.4, 32.6) for adolescents and 29% (95% CI: 25.2, 32.2) for adults.

Exploring how each FOPL format compared with other FOPLs on participants’ ability to identify HFSS, the pattern of results revealed the strongest effect for WL, with the HSR performing worst. Among both adolescents and adults, the WL increased correct identification of HFSS compared with the GDA, the MTL, and the HSR (*P* < 0.05 for all comparisons). Among adolescents and adults, the MTL also increased correct identification of HFSS compared with the GDA and the HSR (*P* < 0.05). Among adolescents and adults, the GDA increased the correct identification of HFSS compared with the control (*P* < 0.05).

### Intentions to purchase

**FIGURE 4:**
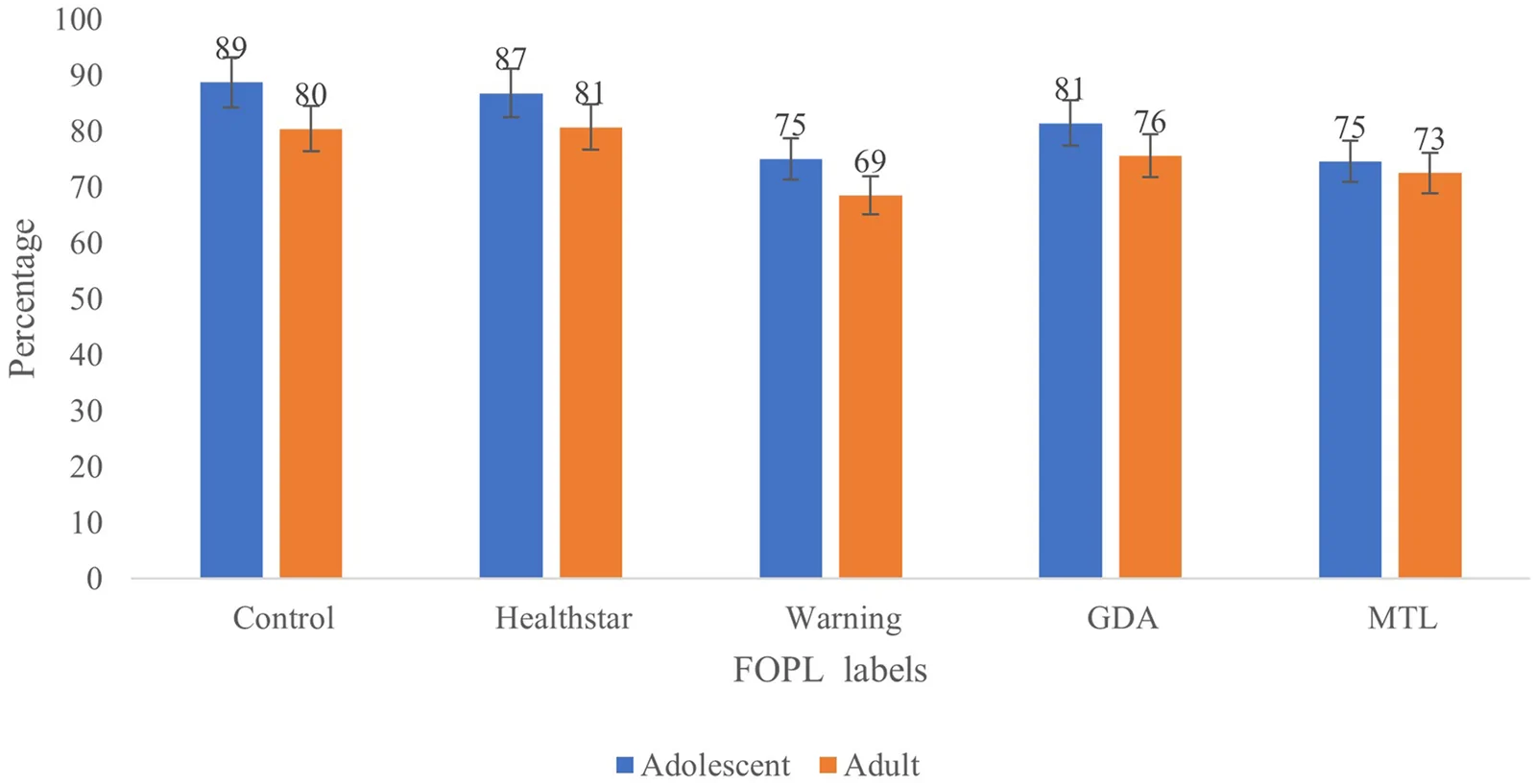
Percentage of participants who intend to purchase the products after intervention.

All FOPL formats significantly reduced intentions to purchase compared with the control (*P* < 0.05 for all comparisons) (Figure 4). HFSS food purchase intentions were highest under the control (adolescents 89%, adults 80%) and HSR labels (adolescents 87%, adults 81%), indicating that these formats exert minimal influence in discouraging product choice. In contrast, interpretive labeling systems, particularly WL (adolescents 75%, adults 69%) and MTL (adolescents 75%, adults 73%), reduced intentions to purchase. The GDA format yielded intermediate effects (adolescents 81%, adults 76%), likely reflecting its reliance on numerical information rather than interpretive cues.

### Secondary outcomes

Table 2 summarizes secondary psychological outcomes among adolescents and adults by FOPL format. All FOPL formats (HSR, WL, GDA, and MTL) produced significantly higher mean scores across all outcomes compared with the control in both age groups (all *P* < 0.001). Among adolescents, WL and MTL showed the highest scores for perceptions of unhealthiness, concern about health problems, PME, and cognitive elaboration. Similar trends were observed among adults, with WL yielding the strongest effects, followed by MTL. Thus, FOPL exposure increased negative health perceptions and message processing relative to no label, with interpretive labels showing stronger effects.

**TABLE 2:** Secondary outcomes for adolescents and adults: average psychological reactions

	Control mean (95% CI)	HSR mean (95% CI)	*P* value^1^	WL mean (95% CI)	*P* value^1^	GDA mean (95% CI)	*P* value^1^	MTL mean (95% CI)	*P* value^1^
**Adolescents**									
Perceptions of unhealthiness	1.7 (1.7, 1.8)	2.2 (2.1, 2.3)	<0.001	3.0 (2.9, 3.0)	<0.001	2.7 (2.6, 2.7)	<0.001	2.9 (2.8, 3.0)	<0.001
Attention	3.1 (3.1, 3.2)	3.7 (3.6, 3.7)	<0.001	3.8 (3.8, 3.9)	<0.001	3.8 (3.7, 3.8)	<0.001	3.8 (3.8, 3.9)	<0.001
Concern about health problems	1.3 (1.2, 1.4)	2.3 (2.2, 2.4)	<0.001	3.2 (3.1, 3.3)	<0.001	2.7 (2.6, 2.8)	<0.001	3.1 (3, 3.2)	<0.001
Seem Unpleasant	1.2 (1.1, 1.3)	2 (1.9, 2.1)	<0.001	2.9 (2.8, 3.0)	<0.001	2.4 (2.3, 2.5)	<0.001	2.7 (2.6, 2.8)	<0.001
Discouraged consumption	1.2 (1.1, 1.3)	2.1 (2, 2.2)	<0.001	3.0 (2.9, 3.1)	<0.001	2.5 (2.4, 2.6)	<0.001	2.8 (2.7, 2.9)	<0.001
PME^2^	1.2 (1.2, 1.3)	2.1 (2.1, 2.2)	<0.001	3 (2.9, 3.1)	<0.001	2.5 (2.5, 2.6)	<0.001	2.8 (2.8, 2.9)	<0.001
Cognitive elaboration	1.4 (1.3, 1.5)	2.4 (2.3, 2.5)	<0.001	3.2 (3.2, 3.3)	<0.001	2.8 (2.8, 2.9)	<0.001	3.2 (3.1, 3.2)	<0.001
**Adults**									
Perceptions of unhealthiness	1.9 (1.8, 1.9)	2.3 (2.2, 2.4)	<0.001	3.0 (2.9, 3.1)	<0.001	2.7 (2.6, 2.7)	<0.001	2.9 (2.8, 3.0)	<0.001
Attention	3.2 (3.1, 3.2)	3.7 (3.6, 3.7)	<0.001	3.8 (3.8, 3.9)	<0.001	3.7 (3.7, 3.8)	<0.001	3.8 (3.8, 3.9)	<0.001
Concern about health problems	1.3 (1.2, 1.4)	2.4 (2.3, 2.4)	<0.001	3.3 (3.2, 3.3)	<0.001	2.8 (2.7, 2.9)	<0.001	3.1 (3.0, 3.2)	<0.001
Seem Unpleasant	1.2 (1.1, 1.3)	2.1 (2.0, 2.2)	<0.001	2.9 (2.8, 3.0)	<0.001	2.4 (2.4, 2.5)	<0.001	2.8 (2.7, 2.9)	<0.001
Discouraged consumption	1.3 (1.2, 1.4)	2.1 (2.0, 2.2)	<0.001	3.0 (2.9, 3.1)	<0.001	2.6 (2.5, 2.7)	<0.001	2.9 (2.8, 3.0)	<0.001
PME^2^	1.3 (1.2, 1.4)	2.2 (2.1, 2.3)	<0.001	3.1 (3, 3.2)	<0.001	2.6 (2.5, 2.7)	<0.001	2.9 (2.9, 3.0)	<0.001
Cognitive elaboration	1.3 (1.3, 1.4)	2.5 (2.4, 2.6)	<0.001	3.3 (3.2, 3.4)	<0.001	2.8 (2.8, 2.9)	<0.001	3.2 (3.1, 3.2)	<0.001

All outcomes are measured on a Likert scale from 1-4, with 1 representing low (not at all) and 4 representing high.

Abbreviations: CI, confidence interval; GDA, guideline daily allowance; HSR, health star rating; MTL, multiple traffic light; PME, perceived message effectiveness; WL, warning label.

1 Each *P* value is for the comparison of FOPL vs. control.

2 PME is the mean of 3 items (Concern about health problems, Seem Unpleasant and Discouraged consumption).

### Tertiary outcomes

Across both adolescents and adults, all FOPL formats performed substantially better than the control across all tertiary outcomes (all *P* < 0.001) (Table 3). Interpretive labels (WL and MTL) consistently showed the strongest responses for almost all tertiary outcomes, followed by GDA and HSR. Compared with the control, exposure to any FOPL format markedly increased perceived label understanding, learning something new, perceived truthfulness, and desire to have the label on products. WL was most frequently identified as the label that discouraged consumption and elicited the highest levels of health concern and perceived unpleasantness, with MTL showing similar but slightly lower effects. These patterns were highly consistent across adolescents and adults, indicating robust effects of interpretive labeling on attention, comprehension, and perceived risk.

**TABLE 3:** Tertiary outcomes by front-of-package labeling arms among adolescents and adults

	Control percent(95% CI)	HSR percent(95% CI)	*P* value^1^	WL percent(95% CI)	*P* value^1^	GDA percent(95% CI)	*P* value^1^	MTL percent(95% CI)	*P* value^1^
**Adolescents**									
Label understanding	33.1 (29.4, 36.9)	83.4 (80.2, 86.1)	<0.001	97.9 (96.5, 98.8)	<0.001	92.2 (89.8, 94.0)	<0.001	97.6 (96.0, 98.5)	<0.001
Learning something new	35.7 (32.0, 39.6)	83.5 (80.4, 86.3)	<0.001	98.1 (96.6, 98.9)	<0.001	93.1 (90.9, 94.9)	<0.001	98.1 (96.6, 98.9)	<0.001
Label seems true	52.7 (48.7, 56.6)	85.7 (82.6, 88.2)	<0.001	97.9 (96.5, 98.8)	<0.001	94.4 (92.3, 96.0)	<0.001	99.2 (98.1, 99.7)	<0.001
Want the label on product	48.3 (44.3, 52.2)	82 (78.8, 84.9)	<0.001	96.0 (94.1, 97.3)	<0.001	92.8 (90.5, 94.6)	<0.001	98.5 (97.2, 99.2)	<0.001
Which label most discourages consumption	9.2 (7.1, 11.7)	50.6 (46.6, 54.5)	<0.001	80.8 (77.5, 83.7)	<0.001	62.3 (58.4, 66.0)	<0.001	74.8 (71.2, 78.0)	<0.001
Grab attention	81.2 (77.9, 84.1)	98.8 (97.6, 99.4)	<0.001	98.7 (97.5, 99.4)	<0.001	99.2 (98.1, 99.7)	<0.001	99.2 (98.1, 99.7)	<0.001
Make concerned about the health consequences.	12.3 (9.9, 15.1)	57.3 (53.4, 61.2)	<0.001	85.6 (82.6, 88.1)	<0.001	69.3 (65.6, 72.8)	<0.001	82.7 (79.5, 85.5)	<0.001
Seem unpleasant	9.8 (7.7, 12.4)	45.1 (41.2, 49.1)	<0.001	76.0 (72.5, 79.2)	<0.001	58.5 (54.6, 62.3)	<0.001	68.3 (64.5, 71.8)	<0.001
**Adults**									
Label understanding	33.8 (30.1, 37.6)	80.7 (77.5, 83.6)	<0.001	95.2 (93.2, 96.6)	<0.001	87.8 (85.0, 90.2)	<0.001	96.8 (95.0, 97.9)	<0.001
Learning something new	49.1 (45.2, 53.0)	85.7 (82.7, 88.2)	<0.001	96.4 (94.7, 97.6)	<0.001	89.3 (86.6, 91.5)	<0.001	97.3 (95.6, 98.3)	<0.001
Label seems true	42.8 (39.0, 46.7)	80.9 (77.6, 83.8)	<0.001	95.5 (93.5, 96.9)	<0.001	88.8 (86.0, 91.1)	<0.001	97.9 (96.4, 98.8)	<0.001
Want the label on product	10.7 (8.5, 13.4)	50.1 (46.2, 54.0)	<0.001	81.1 (77.8, 84.0)	<0.001	65.9 (62.0, 69.6)	<0.001	78.0 (74.6, 81.1)	<0.001
Which label most discourages consumption	15.0 (12.4, 18.1)	64.0 (60.2, 67.7)	<0.001	90.6 (88.1, 92.7)	<0.001	77.1 (73.6, 80.3)	<0.001	87.7 (84.9, 90.1)	<0.001
Grab attention	85.1 (82.1, 87.7)	99.4 (98.3, 99.8)	<0.001	99.5 (98.5, 99.8)	<0.001	99.8 (98.8, 100)	<0.001	99.5 (98.5, 99.8)	<0.001
Make concerned about the health consequences	12.3 (9.9, 15.1)	58.3 (54.3, 62.1)	<0.001	87.4 (84.5, 89.8)	<0.001	71.7 (67.9, 75.1)	<0.001	85.0 (81.9, 87.6)	<0.001
Seem unpleasant	10.0 (7.9, 12.6)	47.5 (43.6, 51.4)	<0.001	77.1 (73.6, 80.2)	<0.001	60.8 (56.8, 64.6)	<0.001	73.2 (69.6, 76.5)	<0.001

Abbreviations: CI, confidence interval; GDA, guideline daily allowance; HSR, health star rating; MTL, multiple traffic light; WL, warning label.

1 Each *P* value is for the comparison of front-of-package label vs. control.

### Sociodemographic differences in correct identification under FOPL

Supplemental Tables 3 and 4 show that all 4 FOPLs (HSR, WL, GDA, and MTL) significantly improved correct identification of products high in nutrients of concern compared with the control across nearly all sociodemographic subgroups (*P* < 0.001). Among all labels, WL consistently demonstrated the strongest performance in both adolescents and adults, in product identification, followed by MTL. Among adolescents and adults, WL remained almost equally effective across both sexes. Regarding the area of residence, rural adolescents demonstrated slightly higher WL effectiveness than urban peers (74.7% compared with 69.5%), whereas the pattern reversed among adults. In urban adult residents, the WL was substantially more effective than the control (72.5% in WL compared with 18.6% in control, *P* < 0.001), whereas in rural residents, the WL was slightly less effective (65.2% in WL compared with 12.4% in control, *P* < 0.001).

In our additional stratified models using 0 to 4 y of schooling as a proxy for illiteracy, we found that participants with 0 to 4 y of schooling consistently showed similar patterns of findings as those with ≥5 y of schooling (Supplemental Table 5).

### RR between the different FOPLs

Supplemental Figures 3 and 4 illustrate the relative likelihood (unadjusted relative risk, RR) of correctly identifying nutrients of concern (e.g., high sugar, sodium, saturated fat, or calories) across different FOPL systems. An RR >1.0 indicates a higher probability of correct identification than the reference label, which therefore represents the less effective format. Cluster-adjusted weighted analysis (Supplemental Figures 5 and 6) showed similar findings (<5% variation in RR for most cases) and the direction of association as well as the statistical significance levels remained unchanged. This illustrated the negligible effect of cluster on the outcome estimates. The confounders (age, sex, area of residence, education, and financial condition) adjusted models produced comparable estimates for almost all the cases (Supplemental Tables 6–8).

#### WL compared with GDA

Across all products and nutrients, WLs were associated with substantially higher relative risks of correct identification (RR > 1.0). For example, adolescents were more likely to correctly identify high-sugar content in cake (RR: 1.24; 95% CI: 1.17, 1.31), and adults were more likely to identify high-saturated fat in salty biscuit (Olympia Lexus) (RR: 1.27; 95% CI: 1.18, 1.37). These findings indicate that WLs are markedly more effective than GDA labels in enabling both adolescents and adults to recognize nutrients of concern.

#### MTL compared with GDA

The MTL system consistently showed higher RRs of correct identification across products. For instance, adolescents were more likely to identify high-sugar content in cake (RR: 1.17; 95% CI: 1.10, 1.24), and adults were more likely to identify high-saturated fat in salty biscuit (Olympia Lexus) (RR: 1.19; 95% CI: 1.10, 1.28). These results suggest that the color-coded, interpretive nature of MTL labels facilitates better understanding than numerical GDA labels. However, the magnitude of effect was generally smaller than that observed for WLs, indicating that although MTLs are more effective than GDAs, WLs remain the most effective approach for communicating nutrients of concern.

#### WL compared with MTL

The WL system has a small advantage over MTL among adolescent consumers. The relative risk is >1.0, indicating that adolescents exposed to WL had a higher probability of correctly identifying certain products with excessive nutrients of concern than those exposed to MTL labels. Only in the case of high-sugar (cake and sweet biscuit) RRs did not touch 1.00, which means WL shows more probability of correctly identifying excessive nutrients of concern than those exposed to MTL labels in these products. For example, identification of high sugar in cake RR [95% CI: 1.06 (1.02, 1.11)] and high sugar in sweet biscuits RR [95% CI: 1.04 (1.01, 1.08)]. In practical terms, this means the simplistic, salient, and negatively framed nature of WL (e.g., a black stop signs) is more effective for this age group than the color-coded MTL system.

Similar to the adolescent results, Supplemental Figure 3B shows that WL and MTL labels have a relatively same effectiveness for adult consumers. The data show that adults using WL had only a bit higher relative likelihood of correctly identifying high-saturated fat [RR 95% CI: 1.07 (1.01, 1.14)] in salty biscuit (Olympia Lexus) than those using MTL.

## Discussion

This randomized controlled trial provides robust evidence on the comparative effectiveness of FOPL systems in Bangladesh, demonstrating that all 4 tested formats, including WL, MTL, GDA, and HSR, significantly improved consumers’ ability to identify foods high in nutrients of concern (sodium, saturated fat, and sugar) compared with no label. Among these, WL consistently emerged as the most effective FOPL system, followed by MTL, with findings remaining stable across age groups (adolescents and adults) and sociodemographic characteristics. Similarly, FOPL formats significantly improved psychological responses compared with the control in both adolescents and adults. WL, followed by MTL produced the strongest effects on perceived unhealthiness, health concern, PME, and cognitive elaboration. In addition, WL and MTL showed the greatest effects on perceived understanding, learning, credibility, and support for labeling, with WL most strongly discouraging consumption and increasing health concern. These results underscore the importance of interpretive, simplified labeling approaches in improving consumer understanding within the Bangladeshi context.

Across all product categories, WLs demonstrated the highest effectiveness in enabling accurate identification of unhealthy foods. Their superior performance relative to other FOPL formats was observed among both adolescents and adults, with particularly pronounced effects among adolescents. MTL labels also improved understanding compared with noninterpretive, numerical formats; however, their effectiveness was generally lower than that of WLs. In contrast, GDA labels consistently performed worst, likely reflecting the higher cognitive burden associated with interpreting numerical information and reference intake values. These findings align closely with evidence from randomized and experimental studies conducted in diverse settings, including India [16], South Africa [17,23], and Chile [24], all of which report superior performance of warning-style labels in supporting consumer understanding of unhealthy food products.

The findings of this study demonstrate that FOPL significantly enhances consumers' ability to correctly identify unhealthy products across diverse sociodemographic groups, with WL emerging as the most effective format consistently outperforming other label types. This aligns with growing evidence from LMIC contexts [17,23], where simple, interpretive labels are more effective than nutrient-specific formats such as GDA. Regarding geographic differences, rural adolescents responded slightly more favorably to WL than their urban peers, suggesting that the binary simplicity of WLs may transcend literacy and socioeconomic barriers prevalent in rural settings. However, this pattern reversed among adults, where urban residents outperformed rural counterparts across nearly all label types, including WL and HSR, which presumably reflects greater exposure to packaged food environments and relatively higher nutritional awareness in urban areas. A mass media campaign, especially focused on rural populations, would be valuable to help ensure the WLs work for those citizens. The differences in FOPL effectiveness between adolescents and adults were small. WL was effective in correctly identifying the nutrients of concern for both adolescents and adults.

The stratified analyses of intervention effects by sociodemographic characteristics did not contradict or amplify the main findings, which consistently showed that interpretive labels were more effective. Our subgroup analyses demonstrated that participants showed a similar pattern in the correct identification of products high in nutrients of concern across educational levels. Thus, interpretive FOPL (WLs) remained more effective than GDA labels across both groups. This association likely reflects the ability of individuals with minimal formal education to process and interpret nutrition information. Policy measures should ensure that FOPLs are designed with simple pictorial cues and supported by public education initiatives so that individuals with no schooling can interpret them effectively. The inclusion of this subgroup was important for representativeness, but the relatively smaller sample size may have limited statistical power to detect differences, and these findings should therefore be interpreted with caution. It is also important to note that our classification of education level was based solely on years of schooling, rather than direct measures of functional literacy. Future studies could incorporate cognitive tests of FOPL interpretation to more precisely capture literacy-related differences in comprehension. Moreover, mixed-methods and robust research could be considered to identify which FOPL formats have the strongest potential to improve food choices among individuals with no schooling or who are functionally illiterate.

Interpretive labels, particularly WL followed by MTL, outperformed noninterpretive formats in enhancing attention, comprehension, and behavioral intent. The higher performance of WL among adolescents aligns with previous evidence showing that direct risk-oriented messaging elicits higher understanding and stronger emotional responses among younger consumers [16,17]. These findings are consistent with the WHO’s recommendation that WL serve as a clear, accessible, and effective tool for reducing the consumption of unhealthy foods [13]. The features of WL might give some sort of advantage to the consumers. It reflects simplified and interpretive design, which focuses exclusively on nutrients present in excess and thereby reduces informational overload on food packaging. Unlike GDA labels, which require consumers to interpret numerical values and reference daily intake benchmarks, WL provide direct and explicit signals of unhealthfulness, enabling faster and more accurate judgments [17,25]. Although MTL labels also offer interpretive prompts through color coding, their correct interpretation still requires an understanding of the color–nutrient relationship, which may limit effectiveness in settings with varying levels of nutrition literacy. In addition, rural-urban comparison showed significantly higher performance for WL and MTL than GDA. WL was comparatively higher among rural adolescents and urban adults. Moreover, evidence from international studies similarly suggests that WL are effective across children [26], adolescents [27], and adults [17], and across educational strata, underscoring their potential to promote equitable access to nutrition information.

The improvements observed in secondary psychological outcomes across all FOPL formats indicate that FOPL effectively enhances consumers’ cognitive and affective responses to unhealthy foods. In both adolescents and adults, WL followed by MTL produced stronger perceptions of unhealthiness, greater concern about health consequences, higher PME, and deeper cognitive elaboration compared with no label, consistent with previous evidence [17,28]. The superior performance of interpretive labels, particularly WL, supports behavioral theories suggesting that simple and salient evaluative cues facilitate rapid risk appraisal and message processing more effectively than noninterpretive formats [13]. The similar pattern of effects among adolescents highlights the potential of FOPL to shape early risk perceptions. These findings have important policy implications for LMICs such as Bangladesh, where rising diet-related NCDs and limited nutrition literacy necessitate low-cost, scalable interventions. Adoption of clear, interpretive FOPL, especially WL, could strengthen consumer understanding and support healthier food environments.

Additional outcomes further substantiate the effectiveness of FOPL as a nutrition communication tool. Across both age groups, interpretive labels enhanced perceived understanding, facilitated learning, and enhanced credibility, attention, and concern regarding health consequences. Moreover, products displaying FOPL were more frequently perceived by participants as discouraging consumption, i.e, the presence of interpretive labeling not only conveyed nutritional information but also acted as a deterrent and potentially reducing the appeal of such products. These findings are consistent with prior experimental and real-world evidence showing that interpretive formats enhance risk perception and message processing more effectively than noninterpretive labels [28]. Similar patterns have been reported in India [16], where WLs improved identification of unhealthy products and reduced purchase intentions, supporting the relevance of interpretive FOPL approaches across diverse settings.

Given the rapid growth of packaged food consumption in Bangladesh and the low salience of existing back-of-pack labels, the introduction of mandatory front-of-pack WLs represents a cost-effective, population-level strategy to improve consumer understanding and help curb diet-related NCDs [1,8]. This approach aligns with the Bangladesh Food Safety Authority’s observation that current labeling practices rarely attract consumer attention [11]. The national representativeness of this study, with balanced inclusion of adults and adolescents, enhances the generalizability of the findings and provides insight into intergenerational differences in label interpretation. As dietary habits formed during adolescence often persist into adulthood [29], effective FOPL may play a critical role in promoting healthier food choices among this group, addressing a growing public health concern in Bangladesh [4,5,7].

The findings of this study have important implications for nutrition policy, public health practice, and food system regulation in Bangladesh. The clear superiority of interpretive FOPL systems—particularly WLs—in enabling both adolescents and adults to correctly identify foods high in nutrients of concern provides strong, context-specific evidence to inform national FOPL policy development. These results suggest that adopting WLs could be an effective, low-cost regulatory intervention to improve consumer understanding of unhealthy foods and support healthier purchasing decisions at the population level.

The stronger performance of WL among adolescents is especially relevant for Bangladesh, where young people are increasingly exposed to packaged foods as part of the ongoing nutrition transition [5]. Implementing simple, salient, and easily interpretable labels may help protect this vulnerable group by reducing cognitive demands and supporting rapid, informed choices. For adults, the comparable effectiveness of WLs and MTL labels indicates that interpretive formats more broadly are preferable to numerical systems, such as GDA, which require higher levels of nutritional literacy.

From a policy perspective, these findings provide strong, context-specific evidence to guide the development of an evidence-based FOPL policy in Bangladesh. The clear superiority of WLs in enabling both adolescents and adults to correctly identify foods high in nutrients of concern supports their adoption as a central component of national nutrition policy. Moreover, FOPL implementation should be integrated into broader strategies to address diet-related NCDs, including consumer education, food reformulation, and systematic monitoring of the packaged food supply. Overall, this study offers timely and locally generated evidence to support the introduction of effective front-of-pack WLs as a key public health intervention in Bangladesh.

### Strengths and limitations of the study

This study’s coverage of all 8 administrative divisions of Bangladesh improves generalizability. To our knowledge, this is the first study to evaluate FOPL effects among adolescents alongside adults, allowing for direct comparison across age groups. Mock food products reduced brand-related bias and isolated label format effects. Several drawbacks should be considered. The investigation was confined to packaged snacks; therefore, future studies should evaluate more food categories to improve external validity. Additionally, long-term studies are needed to evaluate whether FOPLs enhance diet and health over time. Because of time and resource constraints, the number of rural and urban locations was smaller than expected; however, both adolescents and adults met their predefined sample sizes, providing sufficient statistical power and balance across experimental arms. This slight decrease in geographic coverage is unlikely to have affected internal validity, and the study’s findings are robust because of consistency across sociodemographic subgroups.

In conclusion, the present study demonstrates that interpretive FOPL systems substantially improve consumers’ ability to identify nutrients of concern compared with numerical formats. Across study arms, WL consistently showed the highest effectiveness among both adolescents and adults, with a significantly greater likelihood of correctly identifying products high in sugar, sodium, saturated fat, or calories than GDA labels. MTL labels also outperformed GDAs, although their effects were smaller than those observed for WL. Direct comparisons indicate that WLs offer a modest but meaningful advantage over MTL labels among adolescents, particularly for identifying high-sugar products, highlighting the value of simple, salient, and low–cognitive-burden designs for younger consumers. Among adults, WL and MTLs showed broadly similar effectiveness, with only slight advantages for WL in specific nutrients. WL also most effective at reducing consumers’ intention to purchase these harmful products. Overall, these findings support prioritizing WLs as the most effective FOPL approach for enhancing consumer understanding and informing healthier food choices.

## Author contributions

The authors’ responsibilities were as follows –AAS, LST, NS: acquired funding; AAS, LST, LS, SRC, NS: contributed to study design and implementation; OH, NB, MHI, MMH, SS, AKA, UA: coordinated data collection and management; MMH, OH, NB, MHI: performed data analysis; MHI, OH, SS, NB: drafted the manuscript; and all authors: read and approved the final manuscript.

## Data availability

The data supporting the findings of this study are available from the corresponding author upon reasonable request.

## Funding

This research was supported by Bloomberg philanthropy.

## Declaration of generative AI and AI-assisted technologies in the writing process

The author(s) declare that no generative AI or AI-assisted technologies were used in the writing of this manuscript.

## Conflict of interest

The authors report no conflicts of interest.
