# Supplementary material for: Testing Front-of-Package Labels on Packaged Food for Adult and Adolescent Population in Bangladesh
Source: Curr Dev Nutr. 2026 Jul 29;10(9):109464. doi: 10.1016/j.cdnut.2026.109464 (PMC13499218; doi:10.1016/j.cdnut.2026.109464)
Supplement: Supplementary file 1 [file mmc1.docx]

**Supplementary Tables**

Supplementary Table 1: Definition of Different FOPLs

| **Category** | **Multiple Traffic Lights** | **Summary Indicators** | **Reference Intake or Guidelines Daily Amount** | **Warning Label** |
| --- | --- | --- | --- | --- |
| What it is | Traffic lights-like color coding for salt, sugar, fats and saturated fats. Green for low, amber for medium and red for high | Overall rating for nutrition. These are of two types: (i) Health Star (0.5 to 5 rating) (ii) Nutri-score (A to E) | Information on the amount of energy and nutrients, with percentages of the daily reference intake exhausted on consumption as per serve size | Nutrient-specific warnings, conveyed through tools such as colors, shapes and graphics |
| Comment | Can provide conflicting information as a product can be simultaneously red and green. | Not nutrient specific; diabetics and hypertensives cannot know about items high in sugar or salt. Vulnerable to manipulation as the industry can improve score by adding fruit, fibers | Summary of information on negative nutrients at the back of the pack. Too many numbers. Difficult to understand. Industry’s favorite | Easy to understand, gives clear information, the most advanced type interpretive label, current best practice |

Supplementary Table 2: Processed food intake patterns of the participants

|  | **Control**  N = 1230 | **HSR**  N = 1230 | **WL**  N = 1244 | **GDA**  N = 1233 | **MTL**  N = 1237 |
| --- | --- | --- | --- | --- | --- |
| **Adolescent** |  |  |  |  |  |
| **Salty Biscuit Intake** |  |  |  |  |  |
| <1×/week | 478 (78.2) | 481 (79.2) | 496 (79.4) | 492 (78.6) | 488 (79.0) |
| 1×/week | 15 (2.5) | 16 (2.6) | 15 (2.4) | 22 (3.5) | 16 (2.6) |
| >1×/week | 118 (19.3) | 110 (18.1) | 114 (18.2) | 112 (17.9) | 114 (18.4) |
| **Sweet biscuit** |  |  |  |  |  |
| <1×/week | 411 (67.3) | 406 (66.9) | 415 (66.4) | 430 (68.7) | 431 (69.7) |
| 1×/week | 28 (4.6) | 20 (3.3) | 25 (4.0) | 19 (3.0) | 22 (3.6) |
| >1×/week | 172 (28.2) | 181 (29.8) | 185 (29.6) | 177 (28.3) | 165 (26.7) |
| **Cake** |  |  |  |  |  |
| <1×/week | 444 (72.7) | 441 (72.7) | 456 (73.0) | 461 (73.6) | 453 (73.3) |
| 1×/week | 21 (3.4) | 29 (4.8) | 26 (4.2) | 25 (4.0) | 17 (2.8) |
| >1×/week | 146 (23.9) | 137 (22.6) | 143 (22.9) | 140 (22.4) | 148 (23.9) |
| **Chips** |  |  |  |  |  |
| <1×/week | 391 (64.0) | 359 (59.1) | 391 (62.6) | 374 (59.7) | 395 (63.9) |
| 1×/week | 28 (4.6) | 40 (6.6) | 29 (4.6) | 31 (5.0) | 31 (5.0) |
| >1×/week | 192 (31.4) | 208 (34.3) | 205 (32.8) | 221 (35.3) | 192 (31.1) |
| **Chanachur** |  |  |  |  |  |
| <1×/week | 380 (62.2) | 369 (60.8) | 380 (60.8) | 401 (64.1) | 403 (65.2) |
| 1×/week | 33 (5.4) | 37 (6.1) | 37 (5.9) | 31 (5.0) | 24 (3.9) |
| >1×/week | 198 (32.4) | 201 (33.1) | 208 (33.3) | 194 (31.0) | 191 (30.9) |
| **Adult** |  |  |  |  |  |
| **Salty Biscuit Intake** |  |  |  |  |  |
| <1×/week | 497 (80.3) | 500 (80.3) | 491 (79.3) | 489 (80.6) | 480 (77.5) |
| 1×/week | 22 (3.6) | 23 (3.7) | 12 (1.9) | 14 (2.3) | 21 (3.4) |
| >1×/week | 100 (16.2) | 100 (16.1) | 116 (18.7) | 104 (17.1) | 118 (19.1) |
| **Sweet biscuit** |  |  |  |  |  |
| <1×/week | 484 (78.2) | 474 (76.1) | 456 (73.7) | 451 (74.3) | 468 (75.6) |
| 1×/week | 24 (3.9) | 16 (2.6) | 27 (4.4) | 18 (3.0) | 21 (3.4) |
| >1×/week | 111 (17.9) | 133 (21.3) | 136 (22.0) | 138 (22.7) | 130 (21.0) |
| **Cake** |  |  |  |  |  |
| <1×/week | 478 (77.2) | 481 (77.2) | 463 (74.8) | 458 (75.5) | 453 (73.2) |
| 1×/week | 29 (4.7) | 25 (4.0) | 30 (4.8) | 24 (4.0) | 40 (6.5) |
| >1×/week | 112 (18.1) | 117 (18.8) | 126 (20.4) | 125 (20.6) | 126 (20.4) |
| **Chips** |  |  |  |  |  |
| <1×/week | 571 (92.2) | 577 (92.6) | 578 (93.4) | 568 (93.6) | 563 (91.0) |
| 1×/week | 1 (0.2) | 5 (0.8) | 5 (0.8) | 11 (1.8) | 14 (2.3) |
| >1×/week | 47 (7.6) | 41 (6.6) | 36 (5.8) | 28 (4.6) | 42 (6.8) |
| **Chanachur** |  |  |  |  |  |
| <1×/week | 461 (74.5) | 464 (74.5) | 486 (78.5) | 451 (74.3) | 462 (74.6) |
| 1×/week | 37 (6.0) | 25 (4.0) | 20 (3.2) | 28 (4.6) | 32 (5.2) |
| >1×/week | 121 (19.5) | 134 (21.5) | 113 (18.3) | 128 (21.1) | 125 (20.2) |

Note: HSR: Health Star Rating; WL: Warning Label; GDA: Guideline Daily Amount; MTL: Multiple Traffic Light

Supplementary Table 3: Effect of FOPL type on the percent of participants who correctly identified products high in nutrients of concern, moderated by socio-demographic characteristics (Adolescents)

| **Adolescent** | **Control** | **HSR** | **p-value** | **WL** | **p-value** | **GDA** | **p-value** | **MTL** | **p-value** |
| --- | --- | --- | --- | --- | --- | --- | --- | --- | --- |
|  | **% (95% CI)** | **% (95% CI)** |  | **% (95% CI)** |  | **% (95% CI)** |  | **% (95% CI)** |  |
| Gender | | | | | | | | | |
| Boy | 12.9 (9.5, 17.3) | 26.8 (22.1, 32.1) | <0.001 | 73.4 (68.3, 77.9) | <0.001 | 51.9 (46.6, 57.2) | <0.001 | 64.9 (59.2, 70.2) | <0.001 |
| Girl | 14.8 (11.3, 19.2) | 30.8 (25.9, 36.2) | <0.001 | 71.9 (66.5, 76.6) | <0.001 | 55 (49.2, 60.7) | <0.001 | 65.5 (60.2, 70.4) | <0.001 |
| **Age categories** | | | | | | | | | |
| 10-14yrs | 11.6 (8.5, 15.7) | 25.8 (21.2, 31.0) | <0.001 | 68.7 (63.3, 73.5) | <0.001 | 48.3 (42.9, 53.8) | <0.001 | 61.9 (56.5, 67.1) | <0.001 |
| 15-18yrs | 16.3 (12.5, 20.9) | 31.9 (26.9, 37.4) | <0.001 | 76.7 (71.7, 81.1) | <0.001 | 58.7 (53.1, 64.1) | <0.001 | 68.8 (63.3, 73.8) | <0.001 |
| **Education** | | | | | | | | | |
| Grade 0-4 | 8.6 (5.3, 13.8) | 18.2 (13.0, 25.0) | 0.01 | 63.8 (56.0, 70.8) | <0.001 | 38.7 (31.6, 46.3) | <0.001 | 58.8 (50.9, 66.4) | <0.001 |
| Grade-5 | 11.9 (6.9, 19.8) | 24.7 (16.5, 35.2) | 0.027 | 68.6 (59.1, 76.7) | <0.001 | 55.8 (46.1, 65.0) | <0.001 | 51 (41.3, 60.5) | <0.001 |
| Grade 6-9 | 16.8 (12.7, 22.0) | 33.4 (28.3, 39.1) | <0.001 | 77.7 (72.5, 82.2) | <0.001 | 58.5 (52.5, 64.3) | <0.001 | 71.6 (66.2, 76.5) | <0.001 |
| Grade>=10 | 18.6 (11.7, 28.3) | 37.8 (27.5, 49.4) | 0.006 | 77.9 (67.3, 85.8) | <0.001 | 63.1 (52.3, 72.7) | <0.001 | 73 (61.7, 81.9) | <0.001 |
| **Area of residence** | | | | | | | | | |
| Rural | 12.3 (9.3, 16.1) | 28.2 (23.8, 33.0) | <0.001 | 74.7 (70.1, 78.9) | <0.001 | 49.2 (44.1, 54.3) | <0.001 | 64.2 (59.2, 69) | <0.001 |
| Urban | 16.3 (12.2, 21.5) | 29.8 (24.4, 36.0) | <0.001 | 69.5 (63.5, 74.9) | <0.001 | 59.4 (53.3, 65.3) | <0.001 | 66.7 (60.6, 72.2) | <0.001 |
| **Household financial situation** | | | | | | | | | |
| I can meet necessities and buy some luxury items with my income | 9.5 (5.7, 15.4) | 29.3 (22.5, 37.1) | <0.001 | 76.7 (69.2, 82.8) | <0.001 | 56.9 (48.5, 65.0) | <0.001 | 69.8 (62.2, 76.4) | <0.001 |
| I can only meet my necessities with my income | 12.8 (9.7, 16.8) | 30.0 (25.5, 35.0) | <0.001 | 75.5 (70.9, 79.7) | <0.001 | 54.6 (49.5, 59.6) | <0.001 | 67.3 (62.3, 72.0) | <0.001 |
| To meet my necessities, I have to take loans, I cannot buy the necessary stuff | 22.5 (15.9, 30.8) | 24.3 (17.1, 33.3) | 0.75 | 57.0 (47.5, 66.1) | <0.001 | 45.3 (36.5, 54.4) | <0.001 | 50.5 (40.8, 60.1) | <0.001 |

Note: HSR: Health Star Rating; WL: Warning Label; GDA: Guideline Daily Amount; MTL: Multiple Traffic Light; CI: Confidence Interval

Supplementary Table 4: Effect of FOPL type on the percent of participants who correctly identified products high in nutrients of concern, moderated by socio-demographic characteristics (Adult)

| **Adult** | **Control** | **HSR** | **p-value** | **WL** | **p-value** | **GDA** | **p-value** | **MTL** | **p-value** |
| --- | --- | --- | --- | --- | --- | --- | --- | --- | --- |
|  | **% (95% CI)** | **% (95% CI)** |  | **% (95% CI)** |  | **% (95% CI)** |  | **% (95% CI)** |  |
| Gender | | | | | | | | | |
| Man | 12.9 (9.6, 17.1) | 33.2 (28.0, 38.9) | <0.001 | 66.6 (61.3, 71.4) | <0.001 | 50.8 (45.2, 56.5) | <0.001 | 64.1 (58.6, 69.2) | <0.001 |
| Woman | 16.9 13.1, 21.5) | 24.6 (20.3, 29.5) | 0.015 | 70.1 (64.5, 75.1) | <0.001 | 46.8 (41.3, 52.3) | <0.001 | 60.3 (54.7, 65.6) | <0.001 |
| **Age categories** | | | | | | | | | |
| 19-30yrs | 12.8 (9.1, 17.8) | 29.1 (23.6, 35.3) | <0.001 | 75.7 (69.5, 80.9) | <0.001 | 54.1 (47.1, 61.0) | <0.001 | 73.4 (67.5, 78.5) | <0.001 |
| 31-40yrs | 16.2 (11.8, 21.8) | 27.2 (21.5, 33.8) | 0.006 | 69.2 (62.4, 75.3) | <0.001 | 48.3 (41.9, 54.7) | <0.001 | 63.6 (56.6, 70.0) | <0.001 |
| 41-50yrs | 15.4 (10.1, 23.0) | 27.8 (20.9, 36.0) | 0.015 | 62.0 (53.6, 69.8) | <0.001 | 42.7 (34.3, 51.6) | <0.001 | 45.6 (36.7, 54.8) | <0.001 |
| 51-60yrs | 17.3 (9.2, 30.1) | 32.8 (21.9, 45.8) | 0.057 | 53.6 (41.8, 65.0) | <0.001 | 45.5 (32.8, 58.7) | 0.001 | 45.5 (33.9, 57.5) | 0.001 |
| **Education** | | | | | | | | | |
| Grade 0-4 | 10.2 (5.4, 18.5) | 14.4 (8.9, 22.6) | 0.375 | 46.9 (38.4, 55.5) | <0.001 | 18.4 (12.1, 27.1) | 0.101 | 33.9 (25.7, 43.3) | <0.001 |
| Grade-5 | 18.2 (13.2, 24.6) | 27.6 (21.6, 34.6) | 0.033 | 70.8 (63.3, 77.3) | <0.001 | 49.5 (42.6, 56.4) | <0.001 | 61.0 (53.1, 68.4) | <0.001 |
| Grade 6-9 | 11.0 (7.2, 16.7) | 28.6 (22.4, 35.7) | <0.001 | 68.9 (61.4, 75.6) | <0.001 | 51.2 (43.6, 58.8) | <0.001 | 62.9 (55.9, 69.4) | <0.001 |
| Grade>=10 | 17.5 (12.6, 23.7) | 38.7 (31.5, 46.3) | <0.001 | 81.1 (74.4, 86.3) | <0.001 | 67.1 (58.9, 74.4) | <0.001 | 81.5 (74.7, 86.7) | <0.001 |
| **Area of residence** | | | | | | | | | |
| Rural | 12.4 (9.4, 16.1) | 25.9 (21.7, 30.6) | <0.001 | 65.2 (60.2, 69.9) | <0.001 | 46.2 (41.2, 51.3) | <0.001 | 61.5 (56.5, 66.3) | <0.001 |
| Urban | 18.6 (14.2, 24.0) | 32.5 (27.0, 38.6) | <0.001 | 72.5 (66.7, 77.7) | <0.001 | 52.7 (46.4, 59.0) | <0.001 | 63.3 (57.0, 69.1) | <0.001 |
| **Household financial situation** | | | | | | | | | |
| I can meet necessities and buy some luxury items with my income | 9.4 (5.5, 15.5) | 26.6 (20.3, 34.0) | <0.001 | 75.3 (68.1, 81.3) | <0.001 | 50.7 (42.6, 58.7) | <0.001 | 72.0 (64.6, 78.4) | <0.001 |
| I can only meet my necessities with my income | 15.6 (12.3, 19.6) | 29.7 (25.2, 34.6) | <0.001 | 67.6 (62.6, 72.2) | <0.001 | 53.7 (48.5, 58.8) | <0.001 | 62.0 (56.9, 67.0) | <0.001 |
| To meet my necessities, I have to take loans, I cannot buy the necessary stuff | 20.0 (13.1, 29.3) | 27.7 (19.9, 37.2) | 0.203 | 58.1 (47.8, 67.7) | <0.001 | 30.3 (22.4, 39.5) | 0.088 | 48.2 (39.0, 57.5) | <0.001 |

Note: HSR: Health Star Rating; WL: Warning Label; GDA: Guideline Daily Amount; MTL: Multiple Traffic Light; CI: Confidence Interval

Supplementary Table 5: Effect of FOPL type on the percent of participants who correctly identified products high in nutrients of concern, moderated by education label characteristic

|  | **Control** | **HSR** | **p-value** | **WL** | **p-value** | **GDA** | **p-value** | **MTL** | **p-value** |
| --- | --- | --- | --- | --- | --- | --- | --- | --- | --- |
|  | **% (95% CI)** | **% (95% CI)** |  | **% (95% CI)** |  | **% (95% CI)** |  | **% (95% CI)** |  |
| **Adolescents** | | | | | | | | | |
| Grade 0-4 | 8.6 (5.3, 13.8) | 18.2 (13.0, 25.0) | 0.01 | 63.8 (56.0, 70.8) | <0.001 | 38.7 (31.6, 46.3) | <0.001 | 58.8 (50.9, 66.4) | <0.001 |
| Grade ≥5 | 16.0 (12.9, 19.8) | 32.6 (28.4, 37.1) | <0.001 | 75.7 (71.6, 79.4) | <0.001 | 58.7 (54.2, 63.2) | <0.001 | 67.3 (62.9, 71.4) | <0.001 |
| **Adult** | | | | | | | | | |
| Grade 0-4 | 10.2 (5.4, 18.5) | 14.4 (8.9, 22.6) | <0.001 | 46.9 (38.4, 55.6) | <0.001 | 18.5 (12.1, 27.1) | <0.001 | 33.9 (25.7, 43.3) | <0.001 |
| Grade ≥5 | 15.6 (12.8, 19.0) | 31.4 (27.6, 35.5) | <0.001 | 73.7 (69.7, 77.4) | <0.001 | 55.0 (50.6, 59.3) | <0.001 | 68.2 (64.1, 72.1) | <0.001 |

Note: HSR: Health Star Rating; WL: Warning Label; GDA: Guideline Daily Amount; MTL: Multiple Traffic Light; CI: Confidence Interval

Supplementary Table 6: Relative risk between WL and GDA on correct identification of products among adolescents and adults

|  | WL vs GDA (ref)-Adolescent | | WL vs GDA (ref)-Adult | |
| --- | --- | --- | --- | --- |
|  | RR (95% CI) | P value | RR (95% CI) | P value |
| High saturated fat (Olympia Lexus) | 1.14 (1.07, 1.21) | <0.001 | 1.27 (1.18, 1.36) | <0.001 |
| High sodium (Chips, Mr. Chewist) | 1.11 (1.06, 1.17) | <0.001 | 1.16 (1.10, 1.22) | <0.001 |
| High sugar (Cake, Mealtime Family) | 1.24 (1.17, 1.31) | <0.001 | 1.25 (1.18, 1.33) | <0.001 |
| High Sugar (Sweet Biscuits, Mr. Tookie) | 1.13 (1.09, 1.17) | <0.001 | 1.14 (1.09, 1.19) | <0.001 |
| High saturated fat (Sweet Biscuits, Mr. Tookie) | 1.14 (1.08, 1.20) | <0.001 | 1.18 (1.11, 1.26) | <0.001 |
| High Sodium (Chanachur, Shuchi BBQ) | 1.14 (1.09, 1.19) | <0.001 | 1.20 (1.14, 1.27) | <0.001 |
| High saturated fat (Chanachur, Suchi BBQ) | 1.17 (1.11, 1.23) | <0.001 | 1.13 (1.06, 1.20) | <0.001 |

Note: MTL: Multiple traffic light; GDA: Guidelines daily allowance; RR: Relative risk; CI: Confidence interval; the RR was obtained from Poisson regression models adjusted for age, sex, education, area of residence, and financial situation.

Supplementary Table 7: Relative risk between MTL and GDA on correct identification of products among adolescents and adults

|  | MTL vs GDA (ref)-Adolescent | | MTL vs GDA (ref)-Adult | |
| --- | --- | --- | --- | --- |
|  | RR (95% CI) | P value | RR (95% CI) | P value |
| High saturated fat (Olympia Lexus) | 1.10 (1.03, 1.17) | 0.005 | 1.17 (1.09, 1.26) | <0.001 |
| High sodium (Chips, Mr. Chewist) | 1.07 (1.02, 1.13) | 0.005 | 1.09 (1.03, 1.16) | 0.002 |
| High sugar (Cake, Mealtime Family) | 1.17 (1.11, 1.24) | <0.001 | 1.19 (1.11, 1.26) | <0.001 |
| High Sugar (Sweet Biscuits, Mr. Tookie) | 1.08 (1.04, 1.13) | <0.001 | 1.10 (1.05, 1.15) | <0.001 |
| High saturated fat (Sweet Biscuits, Mr. Tookie) | 1.09 (1.03, 1.15) | 0.003 | 1.11 (1.04, 1.19) | 0.002 |
| High Sodium (Chanachur, Shuchi BBQ) | 1.09 (1.04, 1.15) | 0.001 | 1.15 (1.08, 1.21) | <0.001 |
| High saturated fat (Chanachur, Suchi BBQ) | 1.12 (1.06, 1.19) | <0.001 | 1.09 (1.03, 1.16) | 0.004 |

Note: MTL: Multiple traffic light; GDA: Guidelines daily allowance; RR: Relative risk; CI: Confidence interval; the RR was obtained from Poisson regression models adjusted for age, sex, education, area of residence, and financial situation

Supplementary Table 8: Relative risk between WL and MTL on correct identification of products among adolescents and adults

|  | WL vs MTL (ref)-Adolescent |  | WL vs MTL (ref)-Adult |  |
| --- | --- | --- | --- | --- |
|  | RR (95% CI) | P value | RR (95% CI) | P value |
| High saturated fat (Olympia Lexus) | 1.04 (0.98, 1.10) | 0.169 | 1.08 (1.02, 1.15) | 0.01 |
| High sodium (Chips, Mr. Chewist) | 1.04 (0.99, 1.08) | 0.089 | 1.06 (1.01, 1.11) | 0.017 |
| High sugar (Cake, Mealtime Family) | 1.06 (1.02, 1.11) | 0.005 | 1.06 (1.01, 1.11) | 0.02 |
| High Sugar (Sweet Biscuits, Mr. Tookie) | 1.04 (1.01, 1.08) | 0.006 | 1.04 (1.00, 1.08) | 0.081 |
| High saturated fat (Sweet Biscuits, Mr. Tookie) | 1.04 (0.99, 1.09) | 0.091 | 1.06 (1.01, 1.12) | 0.027 |
| High Sodium (Chanachur, Shuchi BBQ) | 1.05 (1.01, 1.09) | 0.027 | 1.05 (1.00, 1.10) | 0.038 |
| High saturated fat (Chanachur, Suchi BBQ) | 1.04 (1.00, 1.09) | 0.06 | 1.03 (0.98, 1.09) | 0.249 |

Note: WL: Warning label; MTL: Multiple traffic light; RR: Relative risk; CI: Confidence interval; the RR was obtained from Poisson regression models adjusted for age, sex, education, area of residence, and financial situation.

**Supplementary Figures**

Supplementary Figure 1: Data Collection locations

Barcode

GDA

Warning

MTL

HSR

Supplementary Figure 2: Five types of FOPLs used in the study (control, MTL, GDA, HSR, WL)

| **A** | **B** |
| --- | --- |
|   **C** |   **D** |

Supplementary Figure 3: Relative risk between the different FOPLs on correct identification of products; A) WL vs GDA (ref) among adolescents, B) WL vs GDA (ref) among adults, C) MTL vs GDA (ref) among adolescents, D) MTL vs GDA (ref)- among adults

| A | B |
| --- | --- |

Supplementary Figure 4: Relative risk between Warning label and Multiple traffic light on correct identification of products; A) WL vs MTL (ref) among adolescents, B) WL vs MTL (ref) among adults

|   **A** |   **B** |
| --- | --- |
|   **C** |   **D** |

Supplementary Figure 5: Relative risk (cluster adjusted) between the different FOPLs on correct identification of products; A) WL vs GDA (ref) among adolescents, B) WL vs GDA (ref) among adults, C) MTL vs GDA (ref) among adolescents, D) MTL vs GDA (ref)- among adults

|   A |   B |
| --- | --- |

Supplementary Figure 6: Relative risk (cluster adjusted) between Warning label and Multiple traffic light on correct identification of products; A) WL vs MTL (ref) among adolescents, B) WL vs MTL (ref) among adults
